# Piloting a Mental Health Literacy Program for Minoritized Adolescent Peer Health Leaders from Under-Resourced Communities

**DOI:** 10.1007/s40596-025-02179-7

**Published:** 2025-07-14

**Authors:** Amira O. Collison, Shiv S. Nadkarni, Naomi C. Wilcox, Shirley A. De La Cruz, Noe Rivera, Maryjane Puffer, Roya Ijadi-Maghsoodi, Enrico G. Castillo, Sheryl Kataoka

**Affiliations:** 1 University of California Los Angeles Semel Institute for Neuroscience & Human Behavior, Los Angeles, CA USA; 2 David Geffen School of Medicine at UCLA, Los Angeles, CA USA; 3 The Los Angeles Trust for Children’s Health, Los Angeles, CA USA; 4 VA Health Service Research (HSR) Center for the Study of Healthcare Innovation, Implementation & Policy (CSHIIP), VA Greater Los Angeles Healthcare System, Los Angeles, CA USA

Addressing the mental health needs of children and adolescents is a major public health challenge in the USA, with one in six youth aged 6–17 experiencing a mental health disorder yearly, and nearly half not receiving care [1]. Access to mental health care for US youth is often hindered by a lack of mental health literacy, which can prevent adolescents and their families from recognizing symptoms or knowing where to seek help. This, combined with systemic issues like provider shortages, insurance limitations, and stigma, leaves many youths unsupported [2]. Consequences of unmet mental health needs in youth can be dire, leading to increased risk for chronic absenteeism and academic failure, substance use disorders, juvenile delinquency, suicide, and unemployment in adulthood [3, 4]. In addition, rates of depression and anxiety have risen substantially among youth in the past decade and have continued to rise since the onset of the COVID-19 pandemic [5].

Black, Indigenous, People of Color (BIPOC) youth have been disproportionately affected by the pandemic due to the exacerbation of pre-existing economic inequities, the disproportionate COVID-19-related morbidity and mortality among families of color, and the challenges associated with online-school-based learning [6]. Youth identifying as BIPOC can additionally face increased levels of psychological stress due to institutional and systemic racism, biases, and socioeconomic disparities that impact their mental health. Due to these and other factors, BIPOC youth remain less likely than their White peers to receive mental health care [7].

One barrier to timely and equitable mental health care in children and adolescents, particularly from under-resourced communities, is mental health literacy. While there are emerging studies evaluating the impact of school-based peer-led mental health interventions on mental health literacy in youth [8], there is a scarcity of such efforts specifically with BIPOC youth from under-resourced communities. Public health education programs in various forms have emerged across US high schools, but school staff may not have the training to deliver mental health education to their students. Academic departments of psychiatry, which often have clinical and research expertise along with a mission to improve the mental health of local communities, can play a unique role in helping to mitigate youth mental health inequities by supporting mental health and wellness education of peer health leaders in public schools. Similarly, these partnerships can equip psychiatry trainees with the necessary skills to meet the needs of diverse populations in community settings. This Educational Case Report describes our pilot program creating an academic-community school mental health partnership and adapting an existing evidence-based high school mental health literacy curriculum for use with BIPOC peer mental health leaders from under-resourced communities. We hypothesized that our community-partnered approach would result in an adapted mental health literacy intervention that is both acceptable and feasible for participating youth and staff. Additionally, we explored whether the intervention would lead to improvements in mental health knowledge, attitudes, and help-seeking behaviors among youth participants.

## Program Implementation and Evaluation

### Peer-Led Mental Health Interventions in Schools

Children spend more time in school than in any other formal institutional structure, making schools an ideal environment for early identification, prevention, and intervention of mental illness. Several studies have demonstrated that mental health interventions in schools are effective in increasing mental health knowledge in youth, although with conflicting data regarding their effectiveness in improving mental health stigma or help-seeking [9]. Peer-led mental health initiatives are emerging as an important mode of delivering health information to youth given there is some evidence to suggest that adolescents are more likely to modify their behaviors and attitudes if they receive health messages from similarly aged peers [10]. The availability of peers from shared identities and ethnic backgrounds offering mental health information may enhance its acceptability among BIPOC students.

### Academic-Community Partnership

Our innovation utilized a community partnered approach, in which all activities including curriculum adaptation, implementation, and evaluation were performed in collaboration with our community partners and student leaders, prioritizing student voices throughout.

The members of our partnership are composed of trainees and faculty at the University of California, Los Angeles (UCLA) Department of Psychiatry and a partnering community-based organization (CBO), named The Los Angeles Trust for Children's Health, which serves a large public school district. The CBO developed a peer-to-peer health education program within the school district, the Student Advisory Boards (SABs). The CBO recruits, trains, and supports the SABs and compensates them for attending regular health trainings and serving as campus health ambassadors. In recent years, SABs reported wanting to learn more about maintaining student well-being and peer mental health challenges. To address this need, we developed an academic-community partnership between the UCLA Department of Psychiatry General Residency Program and the CBO to provide free mental health education and consultation to their staff and student peer health leaders as part of their existing SAB program.

### Curriculum Planning and Adaptation

As a large, urban school district, with 84% of students identifying as Black or Latinx and over 70% qualifying for free/reduced lunch, we adapted the curriculum guide to be culturally responsive to this setting. Our team adapted the *Mental Health & High School Curriculum Guide*, an evidence-based mental health literacy curriculum, for use in the school district. This curriculum was originally designed for high-school aged youth and has been demonstrated to improve mental health literacy among students and teachers [11]. This free, publicly available curriculum guide provides a set of six modules designed to be used by classroom teachers.The topics included are (1) mental health stigma, (2) understanding mental health, (3) specific mental illnesses, (4) experiences of mental illness and importance of family communication, (5) seeking help and finding support, and (6) the importance of positive mental health. These modules were combined and expanded to create the adapted curriculum in Table 1.

**Table 1:** Topics and learning objectives covered in adapted mental health literacy curriculum

Adapted curriculum topics	Adapted learning objectives	Curriculum adaptation
Stress & Stress Management^1^	- Discuss the mind and body response to stress	- Included cognitive-behavioral therapy approach for stress management
	- Learn skills to manage stress in healthy ways to develop resilience	- Reviewed several grounding techniques to reduce stress, including: 5-4-3-2-1, box breathing, and progressive muscle relaxation
	- Learn practical stress reduction techniques to decrease intensity of stress	- Provided resources to local youth mental health supports
	- Discuss healthy vs unhealthy coping skills	
	- Discuss habits that promote positive mental health	
Self-Love & Body Image^2^	- Learn about body image, body dysmorphia, and the psychological impact that beauty standards can place on teens’ mental health	- Included documentary style videos of teens discussing their experiences with unhealthy relationships with food
	- Learn and apply practical techniques of self-compassion and self-acceptance to enhance one’s self-esteem	- Students completed guided self-compassion worksheets in small group breakouts
	- Learn about the signs and symptoms of various eating disorders	- Discussed BIPOC-specific topics of self-criticism (black hair self-deprecation, masculinity standards) and transforming these into self-compassion
	- Review questions to help identify and support a peer with a suspected eating disorder	
What is Mental Health & Mental Health Stigma^3^	- Learn about mental health and understand the difference between mental health distress and mental illness	- Discussed cases centered around BIPOC teens experiencing mental health challenges
	- Learn about the brain-body connection	- Discussed ways to seek help for mental distress in school, home, and community.
	- Learn ways to help a peer demonstrating warning signs of a mental illness	- Reacquainted students with LA-specific youth mental health resources from Linktree presented earlier
	- Learn about stigma surrounding mental illness, and the impact of stigma on help-seeking	
	- Explore the differences between the myths and realities of mental illness	
	- Discuss ways of overcoming mental health stigma	
Gender, Sexuality & Healthy Relationships^2^	- Define and discuss sex, gender identity, gender expression, and sexual orientation	- New mental health topic not included in original curriculum, based on SAB feedback
	- Define terms associated with sex, gender, and sexuality	- Discussed case of emotional abuse centered on an imagined queer BIPOC teen’s lived experience
	- Define gender self-determination and describe its importance	
	- Review signs of healthy vs unhealthy communication in relationships	
	- Define and teach consent	
Mood Disorders in Teens^4^	- Learn about the most common mood disorders in adolescents/teens (major depression, persistent depressive disorder, bipolar I and II disorders, premenstrual dysphoric disorder, substance-induced mood disorder, and borderline personality disorder)	- Reviewed several clinical case examples of mood disorders in BIPOC and queer adolescents to deepen understanding
	- Learn the ways in which mood disorders in teens can be treated	- Discussed differences between normal sadness vs. clinical mood disorders along with warning signs
Anxiety Disorders in Teens^4^	- Learn about the most common anxiety disorders in adolescents/teens (social anxiety, panic disorder, generalized anxiety disorder, post-traumatic stress disorder, specific phobias)	- Reviewed several clinical case examples of anxiety disorders in BIPOC and queer adolescents to deepen understanding
	- Learn the ways in which anxiety disorders in teens can be treated	- Discussed differences between normal stress vs. clinical anxiety disorders along with warning signs
Substance Use Disorders in Teens^4^	- Learn about substance use in adolescents and the ways in which high-risk substance use can negatively affect mental health and adolescent brain development	- Presented harm reduction approach in discussing substance use, misuse, and treatment
	- Review risk factors for high-risk substance use in teens and tools for prevention	- Presented documentary style videos of teens discussing their lived experiences with substance use disorders
	- Discuss warning signs of substance misuse in teens and ways to help peers	- Discussed substances not included in original curriculum, including synthetic marijuana, vaping, and ADHD prescription misuse
		- Identified causes of substance use and brainstormed alternatives to substance use with students
Mental Health Jeopardy^2^	- Review of all the mental health topics covered throughout the year in a fun game of jeopardy	- New lesson created based on students’ feedback requesting fun and interactive mental health lessons

^1^Topic adapted from Original Curriculum Module 6: The Importance of Positive Mental Health

^2^New Topic not included in Original Curriculum

^3^Topic adapted from Original Curriculum Module 1: The Stigma of Mental Illness and Module 2: Understanding Mental Health and Mental Illness

^4^Topic adapted from Original Curriculum Module 3: Information on specific mental illnesses, Module 4: Experiences of mental illnesses, and Module 5: Seeking help and finding support

In the year prior to intervention implementation (October 2021–June 2022), we met weekly with the student leaders and CBO staff to identify key mental health topics of interest to students and cultural adaptations necessary to tailor delivery for our youth population. Curriculum adaptations included case discussions reflecting the racial, ethnic, and gender diversity of our students. We also included additional mental health topics identified by SAB students as relevant, such as a session on “Gender, Sexuality, and Healthy Relationships.” Cultural adaptations included a discussion of black hair self-deprecation in the “Self-Love and Body Image” session.

Other adaptations were applied to curriculum delivery. The original curriculum was delivered by a teacher in a traditional lecture format, whereas the adapted curriculum, in response to student feedback, was designed to be delivered in an interactive small-group format, led by psychiatry trainees and medical students, to promote dialogue and engagement among students.

### Curriculum Implementation

In the pilot year, our team implemented this adapted mental health curriculum with 78 high school peer health leaders from across ten high schools. Students were offered 1 h of mental health training per month from October to May 2022–2023 as part of the SAB program. Students were not required to attend and could attend any number of non-sequential sessions.

The curriculum was delivered by medical students and psychiatry trainees, with faculty oversight. Prior to each lesson, small group leaders received training on the mental health topic and were provided with a facilitator guide for discussions. Trainings were co-developed with the CBO and school district staff to ensure that we followed all district protocols and procedures, with school staff attending each session. In the pilot year, the mental health literacy intervention was delivered virtually due to COVID-19 restrictions.

To encourage student attendance and participation, each student was entered into a raffle for a $25 non-monetary gift related to positive mental health or equipment required for online-based learning at the end of each session.

### Evaluation

The CBO conducted a 20-min anonymous survey at the beginning and end of the school year with the SAB students, as a part of their regular program evaluation. The survey assessed student demographics, curriculum acceptability, feasibility, and mental health literacy changes. The survey utilized the 5-point Likert Scale for responses; numerical values were assigned to each option. For binary data analysis, responses of “agree” and “strongly agree” were consolidated into a single “agree” category, while “disagree” and “strongly disagree” were combined into a single “disagree” category. Remaining response options were categorized as “disagree,” “don’t know,” and “agree.”" The post-survey included open-ended questions on the curriculum’s helpfulness.

Acceptability was defined as positive attitudes and/or beliefs towards the curriculum and its perceived effectiveness. Feasibility was measured by the number of sessions students attended. The *Knowledge and Attitudes to Mental Health Scales (KAMHS)* survey, an evidence-based measure of mental health literacy in children and adolescents with good psychometric properties (total scale *α* = 0.40–0.64), was used to measure mental health literacy across the following domains: (1) mental health knowledge; (2) mental health attitudes; (3) help-seeking behaviors. Wilcoxon signed rank test was used to estimate changes from pre-test to post-test. The survey language was adapted for cultural relevance and 5th grade readability; all participating students were fluent in English.

## Outcomes

Of the 78 students who participated in the intervention, 51.3% (*n*=40) completed the pre-intervention survey, 42.3% (*n*=33) completed the post-intervention survey, and 20.5% (*n*=13) completed both the pre- and post-surveys. On the pre-test survey, the average age was 16 (range 15–20 years), 87.5% (*n*=35) were female, 85% (*n*=34) of participants identified as Hispanic/Latino, and 15% (*n*=6) identified as Black.

On the post-survey, 93.9% of students reported the adapted mental health literacy curriculum improved their understanding of mental health and various mental disorders. In addition, 96.9% of students indicated they were able to utilize information they learned from the adapted curriculum to talk to their peers about mental health. By the conclusion of the intervention, 93.9% (31/33) of students reported feeling comfortable talking about mental health with others and 90.9% (30/33) reported knowing ways to help other students at school when they feel stressed.

Participants found the adapted mental health literacy curriculum to be acceptable, and they expressed several benefits from participation. Common themes from the open-text responses in the survey included improved understanding of mental health and various mental health disorders, decreased mental health stigma, and ability to talk with peers about mental health and connect them to support. When asked how the intervention could be improved, participants recommended more interactive/hands-on learning activities, more education on coping skills, additional mental health topics, and adjusting the time of the webinars to make attendance more accessible. Participant feedback and sample responses appear in Table 2.

**Table 2:** Open-ended feedback on what was helpful about the adapted mental health curriculum from the SAB student post-intervention survey, 2022–2023

Major theme	Subthemes	Illustrative quotes
Mental health literacy	• Understanding of mental health and various mental health disorders	“I found it very helpful that we received knowledge about mental health that not everyone is told or has access to this information.”
		“I gained more knowledge of certain mental health disorders and insight of what those disorders look like, and how people react.”
		“[The webinars] helped me gain a better understanding of mental health.”
		“I found all of the information given to me helpful as it deepened my understanding about mental health.”
	• Understanding ways to maintain positive mental health	“Me ayudaron mucho a comprender cómo manejar el estrés o el uso de mis emociones.” (They helped me a lot to understand how to manage stress or use my emotions.)
		“I found it helpful that I was able to take notes on the processing of emotions and talk about it in my SAB meetings.”
		“I learned how to manage stress and be a good listener for my peers.”
	• Mental health stigmas/attitudes	“It helped me figure more things out about [mental health] stigma.”
		“Something that I found to be very helpful about the UCLA SAB mental health webinars is how much they went into detail about the different ways that mental health affects a person. Not just the person but also their family.”
Mental health promotion among peers		“I could teach my fellow friends about mental health.”
		“The information was very useful for our own tabling events.”
		“I found that the guest speakers knew that we wanted to help peers, so they provided information about our age group that’ll be helpful.”
		“I loved creating plans for school mental health awareness table events.”
		“We could share our resources to people!”
		“I found the information given directly about mental health helpful. I personally feel that I was able to take in the information and relay it to fellow students at my school site.”
Providing a safe space		“That we get to talk about what we feel and to share our opinions.”
		“What I found helpful was that they did a good job in answering any doubts we had about the topics.”

Participants found participation in the innovation to be moderately feasible. Among students who completed the post-test survey, the median number of sessions attended per person was 4.5, and seven students(20.6%) attended all eight sessions.

Of those with both pre- and post-surveys, there was a significant improvement in self-stigmatizing mental health attitudes (*p*=0.01), but no significant changes were seen in mental health knowledge, public mental health stigma attitudes, or help-seeking from pre- to post-test (*p*>0.05).

## Challenges and Next Steps

A practical challenge in implementation that our team faced was the upfront time commitment required of psychiatry trainees to create and deliver the mental health lessons. To help offset the additional time burden on trainees and to ensure the long-term sustainability of this academic-school mental health partnership, our team has taken steps to integrate it into the university’s psychiatry residency training program. We developed a 1-year non-clinical elective which provides dedicated protected time and educational credit for PGY-3 and PGY-4 psychiatry trainees to engage in this educational and community-focused initiative.

Another challenge was maintaining attendance. To encourage attendance, we offered non-monetary gift incentives to the SABs. However, because sessions were held after school, some lessons conflicted with students’ extracurricular activities. Future evaluations may explore protected classroom time so that all peer health leaders can attend and receive the curriculum.

Some limitations should be noted. This pilot evaluation relied on voluntary survey responses collected by the CBO, resulting in low pre- and post-survey response rates among students — potentially due to survey fatigue, confidentiality concerns, or a general mistrust of data collection processes among BIPOC students. Another limitation is that those who completed the post-evaluation surveys were a self-selecting sample and may have been more likely to report the curriculum as favorable. More rigorous evaluations are needed in the future to determine the effectiveness of this curriculum on such outcomes as stigma reduction and help-seeking.

Overall, this innovation met our stated goals of (1) adapting and implementing a mental health literacy curriculum for use with BIPOC peer mental health leaders through a community-participatory process, and (2) promoting community engagement among psychiatry trainees and medical students.

Given the success of our adapted mental health literacy intervention during its pilot year, our community-partnered team envisions several next steps. First, we plan to increase accessibility and engagement by offering hybrid sessions both in person and virtually. Future sessions will dedicate more time assisting peer health leaders with applying the knowledge and skills they learn in preparing for their peer-to-peer mental health campaigns. Additionally, our team is developing a well-being app, co-designed with the SABs and CBO members, that will include information from the mental health literacy curriculum, along with preventative resources that students can access at any time, from their mobile phones.

Our innovation showed promising results and highlighted the potential of academic-community partnerships—particularly among trainees—to prepare and mobilize BIPOC peer mental health leaders. Our collaborative approach, which promotes engagement, cultural relevance, and community buy-in, was fundamental to its success. Other academic institutions with psychiatry training programs should consider this approach for building community partnerships to train peer mental health leaders in their respective communities and to engage psychiatry trainees in community-based education.

## Data Availability

The data supporting this study are available upon request from the corresponding author and approval by The Los Angeles Trust for Children’s Health.
